# Hatching Induction of Cyst Nematodes in Bare Soils Drenched With Root Exudates Under Controlled Conditions

**DOI:** 10.3389/fpls.2020.602825

**Published:** 2021-01-08

**Authors:** Bruno Ngala, Nicolas Mariette, Mélina Ianszen, Pauline Dewaegeneire, Marie-Christine Denis, Catherine Porte, Christophe Piriou, Emilie Robilliard, Antoine Couetil, Eric Nguema-Ona, Jean-Claude Yvin, Virginie Gobert, Amélie Beury, Anne-Claire Le Roux, Josselin Montarry, Sylvain Fournet

**Affiliations:** ^1^FN3PT/inov3PT, Rue des Champs Potez, Achicourt, France; ^2^IGEPP, INRAE, Institut Agro, Univ Rennes, Le Rheu, France; ^3^Société d’Investissement Légumière et maraîchère de Basse Normandie (SILEBAN), Gatteville le Phare, France; ^4^Centre Mondial de l’Innovation-Roullier, Laboratoire de Nutrition Végétale, Pôle Stress Biotiques, Saint Malo, France; ^5^FN3PT/inov3PT, INRAE-IGEPP, Le Rheu, France

**Keywords:** *Globodera*, *Heterodera*, *in vitro*, *in situ*, hatching, soil depth, encysted eggs, root exudates

## Abstract

Cyst nematodes account for substantial annual yield losses in crop production worldwide. Concerns over environmental and health issues due to the use of chemical nematicides mean alternative sustainable and integrated solutions are urgently required. Hatch induction of encysted eggs in the absence of host plants, i.e., ‘suicide-hatching,’ could be a sustainable alternative in reducing population densities of cyst nematodes in infested soils. Here we examined *in situ* hatching of encysted eggs of *Globodera pallida*, *Heterodera carotae*, and *Heterodera schachtii* at varying soil depths, following exogenous applications of host root exudates in repeated glasshouse experiments. Cysts were retrieved 30 or 43 days post-incubation depending on the nematode species and assessed for hatching rates relative to the initial number of viable eggs per cyst. Hatching of the potato cyst nematode *G. pallida* depended on both soil moisture and effective exposure to root exudates, and to a lesser extent on exudate concentration. The carrot cyst nematode *H. carotae* had over 75% hatched induced by root exudate irrespective of the concentration, with better hatch induction at 20 cm as compared with 10 cm soil depth. Hatching of the beet cyst nematode *H. schachtii* largely depended on the soil moisture level at constant temperature, rather than the type or concentration of root exudates applied. As a conclusion, exogenously applied host root exudates may play a major role in inducing *in situ* hatch of encysted eggs of potato and carrot cyst nematodes in the absence of host plant under favorable soil temperature/moisture conditions. To improve such strategy, the characterization of chemical profiles of the root exudate composition and field validation are currently ongoing.

## Introduction

Cyst nematodes are sedentary root endo-parasites of many plants, causing stunted growth and wilt on their hosts. They are associated with serious damages to a broad range of crops amongst which include potatoes, carrots, beets, brassicas and cereals. These organisms represent a major threat in agriculture, especially species belonging to the genera *Globodera* and *Heterodera* which are among the most devastating crop pests worldwide (Jones et al., 2013). The economic losses associated with cyst nematodes are difficult to estimate. An annual figure of 9% worldwide have been estimated for potato cyst nematodes (PCN) for total potato production (Turner and Subbotin, 2013). Annual excesses of US$1.5 billion have been estimated for soybean cyst nematode in the United States alone (Chen et al., 2001), while cereal cyst nematodes may sometimes exceed 90% yield lost depending on environmental conditions (Nicol et al., 2011).

Effective management of cyst nematodes at a field scale is very challenging considering their soil borne nature coupled with their high survival abilities in the absence of the host plant (Marks and Brodie, 1998). The cyst, which corresponds to the desiccated body of the dead female, can contain hundreds of eggs protected against environmental extremes. Infective juvenile (J2) in their dormant state are contained within encysted eggs and can remain viable for several years (Evans and Stone, 1977), making these pests particularly difficult to eradicate once established in the field (Bairwa et al., 2017). The requirements for more environmentally friendly control methods has led to the ban of the broad-spectrum soil fumigant, methyl bromide in 2005, which used to be a silver bullet against soil borne plant parasitic nematodes and complete phase out from use in developing countries in 2015. Hence, there is an urgent need to develop new effective management strategies.

Upon hatch, infective second stage juveniles (J2) are vulnerable and can only navigate through a limited distance, as they depends entirely on their lipid reserves to locate a suitable host to invade and start feeding (Storey, 1984). In potato cyst nematodes, Robinson et al. (1987) noticed that hatched J2 could not survive for more than 2 weeks. This vulnerability is usually offset in some groups of cyst nematode with a sophisticated synchronization of hatching with the presence of signals from the host root exudates (Masler and Perry, 2018). These host-specific compounds collectively referred to as hatching factors include glycoalkaloids or terpenes such as the Glycinoeclepins in root exudates of kidney beans, responsible for hatch induction of *Heterodera glycines* (Masamune et al., 1982; Fukuzawa et al., 1985a, b). Discrepancies in the hatching response to host root exudates exist among cyst nematodes and have been nicely categorized into four broad groups by Perry (2002). In general, this synchronization is very strict for species with a narrow host range, such as *Globodera* spp., which are restricted to members of the Solanaceae (Subbotin et al., 2010), and *Heterodera carotae* which is restricted to the genera *Daucus* and *Torilis* (Aubert, 1986). By contrast, species like *Heterodera schachtii*, which have a wide host range tend to be less dependent on host root exudates. For the latter, *in vitro* hatching of juveniles can be induced with the presence of water only, although hatching is, however, enhanced in the presence of host root exudates (Masler and Perry, 2018).

Here the idea was thus to exploit the vulnerability of hatched J2 and the specificity of hatching factors to develop a control strategy based on the artificial induction of hatching using natural extracts in the absence of the host plant. This strategy, called ‘suicide hatching’ will thus lead to the death of newly hatched J2 and to the reduction of population levels in the soil (Devine and Jones, 2000; Kushida et al., 2003). This is not a new idea and even less a new strategy. Indeed *in vitro* experiments have already demonstrated high levels of hatching in some cyst nematodes, induced by host root exudates produced in laboratory (Mugniéry and Bossis, 1988; Scholte, 2000). Devine and Jones (2001) also reported significant induction of ‘suicide hatching’ on field population of *Globodera rostochiensis* by the incorporation of tomato root exudates in the soil. Their report also indicated that successful application of the ‘suicide hatching’ technique was dependent on soil type, with best results achieved on sandy soils unlike clay and peaty soils. These observations demonstrated the potential of ‘suicide hatching’ in a sustainable management of cyst nematodes but, despite these promising results, this strategy is still not operational in natural conditions. There have been some advances in the development of tools needed for the characterization of the chemical profiles of hatching factors.

This study thus aimed at: (i) screening a large range of wild or cultivated reference host plants previously reported with satisfactory results of hatching induction, (ii) determine the optimal doses and exposure times that induce the highest hatching *in vitro* and (iii) confirm the efficiency of this control strategy in soil experiments according to the applied doses and soil depth. Three cyst nematode species, *G. pallida*, *H. carotae*, and *H. schachtii*, characterized by different ecological requirements and host ranges were tested.

## Materials and Methods

### Nematode Species

Three cyst nematode species were used in these experiments and include the potato cyst nematode (PCN) *G. pallida*, the carrot cyst nematode (CCN) *H. carotae*, and the beet cyst nematode (BCN) *H. schachtii*. The cyst population for each nematode species was from the same generation produced in glasshouse between 2016 and 2018, and stored at 4°C after extraction for minimum of 3–9 months to break obligate diapause prior to use in experiments. Cyst sizes selected for the experiment ranged from 300 to 450 μm in diameter with special attention to physical damage, thus, all broken or cysts with cracks were discarded.

### Root Exudates Production

The production of root exudates from non-tuberous species was performed by soil leaching according to the protocol described by Widdowson and Wiltshire (1958) with some modifications. Seeds were sawn on cell seedling trays and placed in glasshouse regulated at 21/17°C day/night temperatures, respectively, with a photoperiod of 16 h over 2 weeks to attain the four-leaf stage. Seedlings were then transplanted into individual pots filled with 1 L of sterile soil. The collection of root exudates began 2 weeks post-transplanting and continued at a weekly interval up-to week-six. At each time of root exudates collection, plant pots were allowed for 24 h without application of water before being saturated by slowly pouring tap water from the top of the pot. Following pot saturation (water holding capacity), the pots were each suspended on a 1 L beaker before 100 ml of tap water was added from the top of the pot and allowed over 30 min to leach into the beaker underneath the pot. After 30 min, an additional 100 ml of tap water was added to the pot and allowed for a further 30 min before the content of the beaker (200 ml) was retrieved. For each plant species, root exudates collected at the different times were pooled.

Unlike with seeds, to produce root exudates from tuberous species, tubers previously sprouted over 14 days at 21°C in the dark were suspended on tap water in plastic boxes such that the distance between the tubers and water was approximately 0.5 cm. The close proximity of tubers to water meant that roots produced by the tubers were immediately immersed in water. The setup was placed in the dark at 20°C (±0.5°C) and monitored over 21 days, before the water was collected and adjusted such that one tuber was equivalent to 250 ml.

Each batch of root exudates obtained for each species was homogenized and filtrated through cellulose filters of 0.2 μm pore sizes before storage at −20°C until required for the experiments.

### Selection and Screening of Root Exudates

In order to select the most efficient root exudates for each nematode species, a wide range of cultivated plants or wild related members of the same family able to induce hatching of each nematode species were selected based on both their host status and phylogenetic criteria following previous reports such as the studies by Evans (1983); Mugniéry and Bossis (1988), Franco et al. (1999), or Scholte (2000). In addition, some non-host plants or plants with an unknown host status, but phylogenetically related to the host plants were also selected. Overall, 120 species/cultivars, both wild and cultivated plants, were selected for the three cyst nematode species with, respectively, 62 for *G. pallida* 26 for *H. schachtii* and 32 for *H. carotae*. Seeds were obtained from different European Biological Resource Centers and seed sailors.

The ability of root exudates to induce hatching of encysted eggs of *G. pallida*, *H. carotae*, and *H. schachtii* was evaluated *in vitro* with the aid of 24-well plastic plates. For each set of root exudates, one cyst, representing an individual replicate, was deposited on an adapted 250 μm sieve (Ngala et al., 2014) placed in individual wells. The sieve allowed active passage of hatched juveniles, while enabling the easy transfer of the cyst to new/refreshed hatching solution. One ml of the test root exudate, adjusted to 45% of the crude exudates, was then added in each well and plates were incubated in the dark at 20°C (±0.5°C), with each treatment consisting of 12 replicates (i.e., 12 cysts). The number of hatched juveniles were scored at days 1^1^, 2, 4, 10, 15, and 30 days. At each time of assessment, the sieves with the cyst were rinsed and transferred into new wells containing fresh root exudates. At the end of the experiments, individual cysts were opened to count the number of unhatched viable and non-viable eggs/juveniles in a bid to compute hatching rate (as a proportion of the viable eggs).

Following the screening, two most efficient root exudates were selected for each nematode species based on their level of hatch activation and the speed of hatching when the tested nematode species were exposed to the respective root exudates. In addition to rate of hatch induction, the ease of obtaining the cultivars and the production of root exudates played a central role in the selection process.

### Dose and Exposure Time Experiments

To determine the optimum concentrations of root exudates able to activate maximum hatch of cyst nematodes, *in vitro* hatching experiments were set-up with the root exudates selected in the screening assays for *G. pallida*. The root exudates selected included *Solanum tuberosum* cv. Désirée and Iodéa each diluted to 10, 25, 45, and 70% of the crude exudates as compared with tap water (0%). The experiment was conducted following the same protocol used for the screening of the root exudates with the exception that the assessments for hatched juveniles was done at 4, 7, 9, 11, 15, and 30 days post-incubation. Each test exudate or concentration had six replicates. Preliminary experiments with *H. carotae* and *H. schachtii* revealed that exudate concentration was not an important factor, thus it was not necessary to conduct the concentration test on these cyst nematode species.

To determine the exposure time necessary between root exudates and encysted eggs of cyst nematodes to induce a maximum hatching, *in vitro* hatching experiments were set-up in 24-well plastic plates as described above for the determination of optimum concentration. The different exposure time tested included 0, 4, 7, 9, 11, 15, and 30 days for *G. pallida*, and 0, 1, 2, 4, 10, 15, and 30 days for *H. carotae* and *H. schachtii*, with the concentration of root exudates in both cases adjusted to 30% of the crude root exudates. Therefore, assessments for hatched J2 were conducted at the stated exposure times, during which, cysts were either transferred into water after their respective time of exposure with root exudates, or the solution was refreshed if the end of the exposure time of the cysts with the hatching solution had not finished. Each test exudate or concentration had six replicates.

To determine whether a continuous exposure was needed to induce a maximum hatching, cysts were periodically exposed to root exudates for 4 h every 6 days before re-deposition into tap water. A positive control consisted of a treatment with continuous exposure between root exudates and cysts, while a negative control (continuous exposure with water) were added for each nematode species. The test root exudates were diluted to 85% for *H. schachtii* and 45% for *G. pallida* and *H. carotae*. All treatments had six replicates.

### Root Exudates Efficiency Under Soil Conditions

These experiments were conducted in 2018 and 2019 under controlled environmental conditions (20/16°C day/night temperatures, respectively, with a photoperiod of 16 h) at three different sites depending on the nematode species. Samples of soils used in all experiments were sent to Centre Mondial d’Innovation (CMI) Groupe-Roullier, St-Malo, France for texture and pH analysis.

All experiments except experiments with *H. schachtii* were conducted in plastic pots (35 cm height-8 cm diameter) under controlled conditions, filled with natural field soils free from any cyst nematode species. Smaller plastic pots (8 cm^∗^8 cm^∗^8 cm) were used in experiments with *H. schachtii*.

In all experiments, each set of pot experiments was arranged in a randomized complete block design with each treatment having ten replicates. The soil moisture was maintained around 80% of field capacity throughout the period of test. Therefore, prior to each application of treatment or water, the moisture content of the soils was recorded to determine the amount of water loss. This lose was then compensated by the prior application of tap water to the respective pots with water deficit where applicable to attain 80% of the field capacity.

For the three nematode species, the initial number of encysted viable eggs (*P*_*i*_) was estimated from 10 × 10 cysts. Therefore, 10 cysts from same population measuring between 300 and 450 μm were randomly picked and placed onto a tiny droplet of tap water on a piece of aluminum block and crushed with the aid of a glass slide. The crushed cysts/eggs mixture was rinsed into an 80 mL Pyrex beaker, vigorously homogenized to separate egg clusters and suspended into 40 mL tap water. The suspension was homogenized before aliquots of 3 mL × 1 mL were pulled with a 2 mL glass pipette onto 1 mL nematode counting slide for the quantification of viable and non-viable J2 to determine the initial viable eggs per cyst (*P*_*i*_).

At the end of the experiments, to count the remaining (unhatched) viable J2 per cyst (*P*_*f*_) and determine the hatching rate for each treatment, the incubated cyst sachets were retrieved and numbered according to levels, before being arranged into appropriately labeled petri-dishes according to treatment on each block. Each sachet was carefully cut open with a pair of scissors and the cysts were carefully retrieved. The number of viable and non-viable eggs/cyst was then estimated as described above for *P*_*i*_ assessments. The hatching percentage for each treatment was then computed as the ratio of difference between the mean *P*_*i*_ and *P*_*f*_ values to the mean *P*_*i*_ value ([(*P*_*i*_ *− P*_*f*_)/*P*_*i*_]) and expressed as percentages.

Experiments with *G. pallida* were conducted in 2018 at FN3PT/inov3PT (Achicourt, France) and INRAE (Le Rheu, France) laboratories with soils collected from Laon and La Gruche, respectively, and repeated in 2019 only in FN3PT/inov3PT. Soils were pre-moistened at 45 to 50% of field capacity prior to filling into experimental pots to the 30 cm level. In 2018, treatments included Désirée root exudates at 25% or 45% dilutions of the crude root exudates, with tap water as control. In the repeated experiments in 2019, exudates were further diluted to 5% while maintaining 25% of the crude root exudates with the addition of Iodéa root exudates to the treatment list. Each pot was inoculated with three sachets each containing 10 cysts placed at 5, 15, and 25 cm from top soil. The mesh allowed the movement of hatched J2 from the cysts to the soil. Experimental pots each received 60 ml of the test root exudates at their respective concentrations or tap water at a 4 days interval over the first 16 days (5 applications). Soil moisture was then maintained with tap water only until the end of the experiments (43 days).

Experiments with *H. carotae* were conducted at the SILEBAN laboratories (Gatteville-le-Phare, France). The soil used for the experiments was collected from a single field (Gatteville-le-Phare, France). Pots were filled with soil as describe above for *G. pallida* and inoculated with two sachets each containing 15 cysts at 10 and 20 cm depth from top soil. Treatments for these experiments included carrot cv. Touchon or cv. Pusar Kesar, as crude exudates or at 50% dilution, with tap water as a control. Each pot received 50 ml of the respective treatments or tap water at a 4-days interval for the first 20 days (six applications). We then maintained the moisture content with tap water only until the end of the experiments (40 days).

Experiments with *H. schachtii* were conducted at the INRAE laboratories (Le Rheu, France), with the same soil used in the experiments with *G. pallida*. Treatments for these experiments included undiluted root exudates of sugar beet cv. Acacia or tap water as control. Pots were inoculated with one sachet only, at 4 cm depth from top soil, containing 10 cysts for each replicated treatment. For treatment with root exudates, two application regimes (R) were employed, which included an amount of 15 or 30 ml of undiluted root exudates every 2 (R1) or 4 (R2) days, respectively, during the first 16 days. We then maintained the moisture content with 30 ml of tap water until the end of the experiments (30 days).

### Statistical Analysis

All data was checked for normality of residuals with the aid of the residual and mean plots. Data for pot experiments were subjected to a general analysis of variance (ANOVA) specifically the general treatment structure in randomized blocks using GenStat^®^ 19th Edition software pack (VSNI Products, United Kingdom). Significant differences between treatments and controls were determined using Tukey’s multiple range test (5% significance level). For dose and exposure time experiments, the relationship with the percentage of hatched eggs per cyst was explored using linear and quadratic models. The model selection based on Akaike’s information criteria, AIC (Johnson and Omland, 2004), was used in order to determine the model of best fit. Linear regression was therefore, performed on exposure time experiments to observe the relationship with the rate of hatch for each cyst nematode species, while quadratic models were used to explain dose effect on rate of hatching for *G. pallida*.

## Results

### Root Exudates Screening Experiments

Root exudates collected from 66 plants and/or varieties were screened for *G. pallida* hatch induction: 62 specifically selected for this species and two root exudates each initially selected for *H. carotae* and *H. schachtii* (Table 1A), respectively. High levels of hatch induction was observed for root exudates of some solanaceous plants, with the top five including *Solanum tuberosum* cv. Iodéa & Magnum, *S. gourlayi*, *S. tuberosum* cv. Désirée, and *S. melongena* cv. Listada de Gandia, in this order, which accounted for 75–95% hatch induction after an incubation period of 30 days (Table 1A). The hatching rate was faster for root exudates of some potato varieties such as Iodéa, Magnum, Blanche as well as tomato cv. Saint-Pierre in which between 41 and 79% hatch was observed just after 10 days exposure of encysted eggs of *G. pallida* to the root exudates (Table 1A). Although the percentage hatch of *G. pallida* was generally low in root exudates of non-solanaceous plant species, (<30%), some solanaceous plants also induced very low hatch (Table 1A). Surprisingly, 56% hatch was observed in root exudates of *Brassica oleracea* cv. ‘Early Sprouting,’ a non-solanaceous plant.

**TABLE 1A:** Plant species and/or cultivars screened for the ability of their root exudates to induce hatching of *Globodera pallida in vitro.*

Species	Cultivar	Family	Source	% Hatch (10-*dpe*)	% Hatch (45-*dpe*)
*Solanum tuberosum*	Iodéa	Solanaceae	Tubers	79.0	95
*S. tuberosum*	Magnum	Solanaceae	Tubers	67.0	89
*S. gourlayi*	88S.315.18	Solanaceae	Tubers	22.0	86.6
*S. tuberosum*	Désirée	Solanaceae	Tubers	50.0	80.9
*S. melongena*	Listada de Gandia	Solanaceae	Grains	34.0	75.1
*S. demissum*	69S.167.104	Solanaceae	Tubers	34.9	68.9
*S. tuberosum*	Blanche	Solanaceae	Tubers	43.0	62
*S. tuberosum*	Inovator	Solanaceae	Tubers	35.0	62
*S. viarum*		Solanaceae	Grains	11.1	62
*S. spegazzinii*	88S.334.19	Solanaceae	Tubers	26.2	61.8
*S. andigena*	88S.255. 2	Solanaceae	Tubers	14.8	61.6
*S. tuberosum*	94T146.52	Solanaceae	Tubers	16.8	61.4
*S. vernei*	78S.248. 4	Solanaceae	Tubers	24.8	60.3
*S. phureja*	78S.222. 7	Solanaceae	Tubers	18	58.5
*S. lycopersicum*	Saint-Pierre	Solanaceae	Grains	41	57.5
*Brassica oleracea* var. italica	Early purple sprouting	Brassicaceae	Grains	12.8	56.7
*S. stenotomum*	74S.14.1	Solanaceae	Tubers	21.1	55.7
*S. tuberosum*	Désirée	Solanaceae	Grains	30.7	55.4
*S. sisymbriifolium*		Solanaceae	Grains	24.1	54.0
*S. melanocerasum*		Solanaceae	Grains	20.5	52.9
*S. lycopersicum*	Cornue des Andes	Solanaceae	Grains	32.9	52.5
*S. tuberosum*	Stronga	Solanaceae	Tubers	35	50
*S. berthaultii*	88S.282. 36	Solanaceae	Tubers	20.2	49.1
*S. schenckii*	99S. 72. 6	Solanaceae	Tubers	16.8	42.8
*S. mauritianum*		Solanaceae	Grains	18.6	40.3
*S. aethiopicum*	Var. N’Goyo	Solanaceae	Grains	16.2	39.3
*S. andigena*	88S.249. 1	Solanaceae	Tubers	20.4	39.1
*S. phureja*	88S.511.7	Solanaceae	Tubers	19.6	38.2
*S. pseudocapsicum*		Solanaceae	Grains	15.3	32
*S. trifidum*	00S.100. 20	Solanaceae	Tubers	8.1	29.5
*Ullucus tuberosus*		Basellaceae	Tubers	11.7	28.8
*Nicotiana tabacum*	Xanthi	Nicotianeae	Grains	13.3	28.7
*S. cardiophyllum*	00S. 42. 3	Solanaceae	Tubers	8.1	28.6
*Petunia inflata*		Solanaceae		14.2	27.5
*S. melongena*	Barbentane	Solanaceae	Grains	16.7	26
*Oxalis tuberosa*		Oxalidaceae	Tubers	7	25.7
*S. gourlayi*	88S.495.5	Solanaceae	Tubers	5.3	25.5
*S. tarijense*	90S. 14. 11	Solanaceae	Tubers	16.2	24.6
*S. nigrum*		Solanaceae	Grains	9	22.2
*Foeniculum vulgare*	Cormo	Apiaceae	Grains	8.6	21.9
*S. stoloniferum*	00S. 83. 13	Solanaceae	Tubers	8.3	21.2
*Lupinus* sp.		Fabaceae	Grains	15.1	21
*S. macrocarpon*		Solanaceae	Grains	5.7	19.9
*S. sparsipilum*	88S.329.18	Solanaceae	Tubers	7.8	19.6
*Physalis peruviana*	Peruviana	Solanaceae	Grains	10.7	18.4
*Brugmansia suaveolens*		Solanaceae		6.4	17.2
*Cestrum parqui*		Solanaceae		9.9	16.4
*Ipomoea purpurea*	Royal Ensign	Convolvulaceae	Grains	7.8	14.4
*Orge commune*		Poaceae	Grains	7.4	14
*Ipomée*		Convolvulaceae	Grains	6.6	14
*S. polytrichon*	00S. 69. 8	Solanaceae	Tubers	4.4	13.6
*S. hougasii*	00S. 60. 1	Solanaceae	Tubers	4.8	12.7
*Capsicum annuum*	Yolo wonder	Solanaceae	Grains	2.9	10.8
*N. tabacum*	Benthamiana	Nicotianeae	Grains	5.1	9.8
*S. stenotomum*	74S. 16. 3	Solanaceae	Tubers	3	9.2
*Datura stramonium*		Solanaceae	Grains	3.4	9
*B. juncea*	Aurea	Brassicaceae	Grains	1.9	7.3
*D. carota*	Western red	Apiaceae	Grains	0.6	6.2
*Iochroma australe*		Solanaceae		1.6	5.4
*Ipomoea batatas*		Convolvulaceae	Tubers	0	4.7
*Petunia integrifolia*		Solanaceae	Grains	1.7	4.1
*C. annuum*	Cayenne	Solanaceae	Grains	1.6	3.9
*C. annuum*	Belrubi	Solanaceae	Grains	0	1
*Tropaeolum tuberosum*		Tropaeolaceae	Tubers	0.3	0.7

Root exudates collected from 34 plants were screened for *H. carotae*, with 32 specifically selected for this species and one each initially selected for *G. pallida* and *H. schachtii*, respectively. Results (Table 1B) highlighted two distinct group of plants: the first group induced hatching of 20% and above, and included wild or cultivated carrot varieties and species, whereas the second group had less than 1% hatch induction, and included plants not belonging to the carrot’s family. Among the exudates of the first group, three induced hatching of 70% and above, especially the carrots cv. Touchon and Pusa Kesar (Table 1B).

**TABLE 1B:** Plant species and/or cultivars screened for the ability of their root exudates to induce hatching of *Heterodera carotae in vitro.*

Species	Cultivar	Family	Source	% Hatch (10-*dpe*)	% Hatch (30-*dpe*)
*D. carota*	Touchon	Apiaceae	Seed	8	84.3
*D. c. carota* ssp. *maximus*		Apiaceae	Seed	11.7	72.8
*D. carota*	Puska kaesar	Apiaceae	Seed	27	69.6
*D. carota*	Western red	Apiaceae	Seed	14.3	63.9
*D. c. carota* ssp. *carota* var. *carota*		Apiaceae	Seed	3	63.3
*D. c. gummifer* ssp. *gummifer* var. *gummifer*		Apiaceae	Seed	20.4	58
*D. c. gummifer* ssp. *drepanensis*		Apiaceae	Seed	2.2	55.2
*D. carota*	Haian-3-sun	Apiaceae	Seed	9.6	54.3
*D. carota*	Nantaise	Apiaceae	Seed	5.6	52.4
*D. c. intermediaire*		Apiaceae	Seed	2.8	48.5
*D. c. gummifer* ssp. *hispidus*		Apiaceae	Seed	2	42.2
*D. carotae*	T16	Apiaceae	Seed	2.6	40.9
*D. c. gummifer* ssp. *hispanicus* 684		Apiaceae	Seed	3.7	40.9
*D. carota*	Muscade d’Alger	Apiaceae	Seed	8.5	36.8
*D. c. gummifer* ssp. *commutatus*		Apiaceae	Seed	3	33.2
*D. c. carota* ssp. *maritimus*		Apiaceae	Seed	2.2	33
*D. carota*	Violette turque	Apiaceae	Seed	14.3	31
D. *c. carota* ssp. *gadecaei*		Apiaceae	Seed	3	25.4
*D. carota*	Jaune du Doubs	Apiaceae	Seed	1.7	20.9
*D. capillifolius*		Apiaceae	Seed	0.7	20.7
*Petroselinum crispum*		Apiaceae	Seed	0.4	0.6
*P. c.* ssp. *tuberosum*		Apiaceae	Seed	0,0	0.6
*Pastinaca sativa*		Apiaceae	Seed	0.2	0.2
*Apium graveolens* var. *rapaceum*		Apiaceae	Seed	0.2	0.2
*Foeniculum vulgare*	“Finale”	Apiaceae	Seed	0.0	0.2
*Anthriscus cerefolium*		Apiaceae	Seed	0.0	0.2
*F. vulgare*	“Cormo”	Apiaceae	Seed	0.2	0.2
*Beta vulgaris*	Julietta	Amaranthaceae	Seed	0.2	0.2
*Anethum graveolens*		Apiaceae	Seed	0.0	0.0
*Anthriscus cerefolium crispum*		Apiaceae	Seed	0.0	0.0
*Conium maculatum*		Apiaceae	Seed	0.0	0.0
*Coriandrum sativum*		Apiaceae	Seed	0.0	0.0
*Apium graveolens*		Apiaceae	Seed	0.0	0.0
*S. tuberosum*	‘94T146.52’	Solanaceae	Seed	0.0	0.0

Root exudates collected from 30 plants were screened for *H. schachtii*, with 26 belonging to the Brassicaceae (host family), and two each initially selected for *G. pallida* and *H. carotae* (Table 1C). Plants belonging to the host range induced high hatching levels (above 80%) after 30 days exposure. Sugar beets cv. Julietta and Acacia were notably the most efficient, inducing 100% hatch *in vitro*. The rate of hatch was faster in root exudates from species such as *Brassica carinata* cv. Aurea and *B. oleracea* cv. Early sprouting (Table 1C).

**TABLE 1C:** Plant species and/or cultivars screened for the ability of their root exudates to induce hatching of *Heterodera schachtii in vitro.*

Species	Cultivar	Family	Source	% Hatch (2-*dpe*)	% Hatch (30-*dpe*)
*Beta vulgaris*	Julietta	Amaranthaceae	Seed	63.8	100
*Beta vulgaris*	Acacia	Amaranthaceae	Seed	56.9	100
*Brassica oleracea* var. *botrytis*	Snowball	Brassicaceae	Seed	68.7	100
*B. napus*	Yudal	Brassicaceae	Seed	83.2	99.3
*B. oleracea* var. *italica*	Early purple sprouting	Brassicaceae	Seed	82	97.3
*Amaranthus cruentus*	Golden Giant	Amaranthaceae	Seed	66.7	95.1
*B. juncea*	Aurea	Brassicaceae	Seed	86.5	93.9
*Aurinia saxatilis*		Brassicaceae	Seed	72.1	93.5
*Beta vulgaris*	Bison	Amaranthaceae	Seed	50.9	91.9
*Beta vulgaris*	Sanetta	Amaranthaceae	Seed	54.1	91.4
*B. rapa* ssp. *pekinensis*		Brassicaceae	Seed	79.9	91.4
*Beta vulgaris*	Ardan	Amaranthaceae	Seed	57.6	87.2
*Spinacia oleracea*	Géant d’hiver	Amaranthaceae	Seed	42.4	86.8
*B. napus*	Alpaga	Brassicaceae	Seed	64.3	86.8
*Beta vulgaris*	Nemata	Amaranthaceae	Seed	64.6	86.2
*B. vulgaris* ssp. *maritima*		Amaranthaceae	Seed	30	85.5
*Portulaca oleracea*		Portulaceae	Seed	56.6	84.4
*Atriplex hortensis*		Amaranthaceae	Seed	43	83.6
*Nasturtium officinale*		Brassicaceae	Seed	57	82.9
*S. tuberosum*	Désirée	Solanaceae	Tuber	67	82.3
*Pisum sativum*	James	Fabaceae	Seed	35	82
*Chenopodium quinoa*		Amaranthaceae	Seed	32.3	81.9
*Pastinaca sativa*	Demi Long de Guernesey	Apiaceae	Seed	47.2	79.3
*D. carota* ssp. *sativus*	Nantaise	Apiaceae	Seed	41.1	68.5
*Dianthus barbatus*	Kaléïdoscope	Caryophyllaceae	Seed	42.1	66.6
*A. thaliana*		Brassicaceae	Seed	43.6	64.9
*S. lycopersicum*	“Saint-Pierre”	Solanaceae	Seed	52.3	64.6
*Sinapis alba*	Cador	Brassicaceae	Seed	22	61.1
*Eruca vesicaria* ssp. *sativa*		Brassicaceae	Seed	26	60.3
*Pisum sativum*	Lumina	Fabaceae	Seed	6.3	47.6

Following results from the screening, *Solanum tuberosum* cv. Iodéa and Désirée were selected for *G. pallida*, while *Daucus carota* cv. Touchon and cv. Pusa Kesar were selected for *H. carotae* and *Beta vulgaris* cv. Acacia and was selected for *H. schachtii* (Table 1).

### Dose and Exposure Time Experiments

**FIGURE 1:**
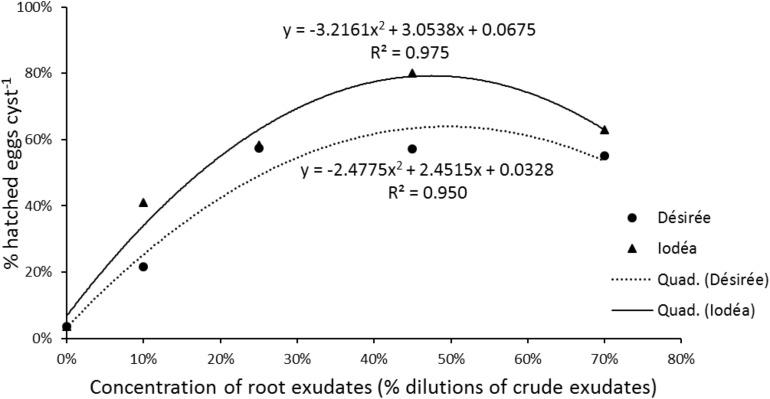
The relationships between *in vitro* hatching of *Globodera pallida* and concentration (0, 10, 25, 45, or 70%) of the crude root exudates of *Solanum tuberosum* var. Désirée or Iodéa. The regression coefficient (*R*^2^) and equations of the quadratic model are indicated for each potato variety. Data points represents means values of six replicates.

For the dose effect, the quadratic model fitted best for the data, for the test root exudates (Désirée an Iodéa). Dose dependent hatch induction of *G. pallida* showed an optimum performance at 45% concentration of root exudates for potato cv. Iodéa. *Globodera pallida* hatch induction by potato cv. Désirée root exudates increase to a peak of 25%, before stabilizing from 25 to 70% concentration (Figure 1). Generally, hatching of *G. pallida* was positively correlated with concentration of Iodéa (*R*^2^ = 0.975; *P* = 0.025) up to 45% concentrations, beyond which the rate of hatching draped. Similarly, Désirée root exudates showed positive relationship with *G. pallida* hatching (*R*^2^ = 0.950; *P* = 0.047) up to a peak of 25% with further increase in dose having no additional effect on% hatch (Figure 1).

**FIGURE 2:**
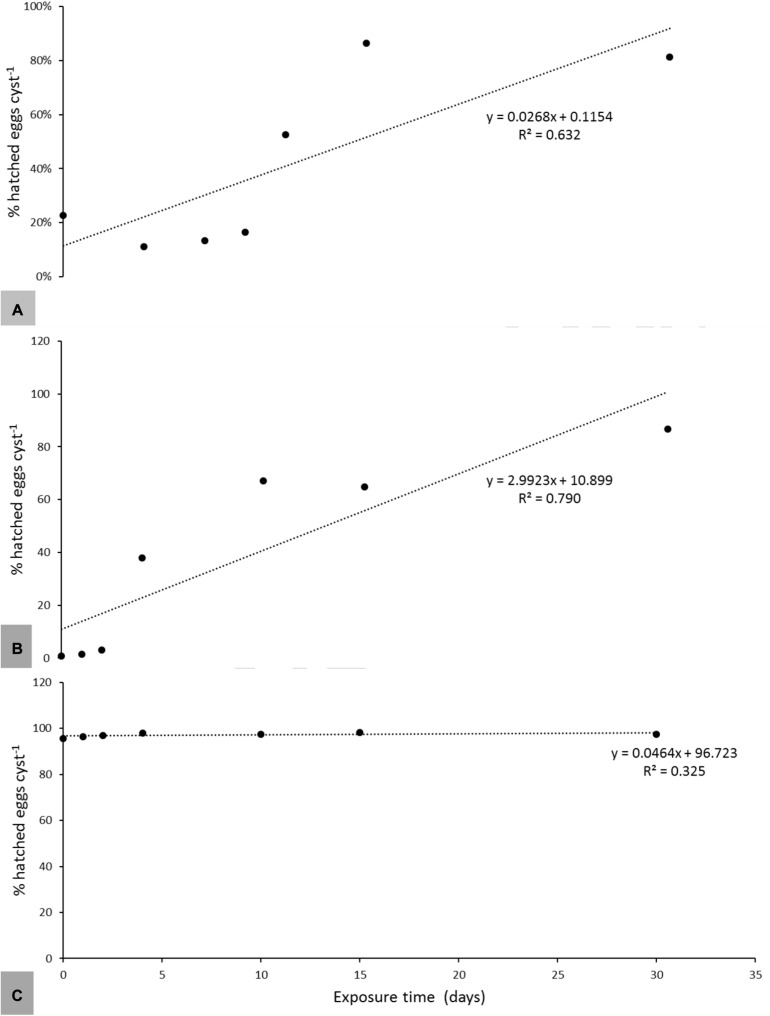
The relationships between *in vitro* hatching of *G. pallida* **(A)**, *H. carotae* **(B)**, and *H. schachtii* **(C)** and exposure time to root exudates of *Solanum tuberosum* var. Désirée, *Daucus carota* cv. Touchon and *Beta vulgaris* cv. Acacia at 45% dilution, respectively. The regression coefficient (*R*^2^) and equations of the linear model are indicated for each potato variety. Data points represents means values of six replicates.

A periodic exposure for 4 h every 6 days induced similar hatching rates in the three species as a continuous exposure to root exudates (Table 2). Linear regression analysis revealed that the exposure time between root exudates and encysted eggs necessary to induce significant levels of hatch was an important factor for *G. pallida* and *H. carotae*, but not for *H. schachtii* (Figure 2). Generally, 11 days exposure to Désirée root exudates was necessary to induce a significant (*R*^2^ = 0.632; *P* < 0.001) amount of hatching of *G. pallida*, with an optimum hatch at 15 days of incubation (Figure 2A). *Heterodera carotae* needed a minimum of 4 days exposure to root exudates for a significant induction (*R*^2^ = 0.790; *P* < 0.001) hatch, with an optimum exposure time of 30 days (Figure 2B). However, *H. schachtii* had a similar level of hatching in water and root exudates, with a weak and non-significant (*R*^2^ = 0.325; *P* = 0.088) relationship with exposure time under *in vitro* conditions (Figure 2C).

**TABLE 2:** Percentage hatched eggs cyst^–1^ (±SE) following continuous or periodic exposure for 4 h every 6 days to root exudates over 30 days.

Nematode species	Root exudate	Dilutions (%)	Exposure time of cysts with root exudates		
			4 h every 6 days	Continuous exposure	No exposure (water)
*Globodera pallida*	Potato var. Désirée	30	91.9 (1.4)^*a*^	95.7 (0.6)^*a*^	14,1 (5.9)^*b*^
*Heterodera carotae*	Carrot cv. Touchon	30	68.3 (6.8)^*a*^	78.8 (5.9)^*a*^	1,1 (0.9)^*b*^
*H. schachtii*	Sugar beet cv. Acacia	85	98.2 (0,1)	93.2 (1.0)	93.9 (1.9)

*Data across each raw with similar superscript lower case letters are not statistically different according to Tukey test (5% level of significance).*

### Root Exudates Efficiency Under Soil Conditions

An analysis for the particle size distribution of the soils used for the pot experiments revealed that the soil texture was more or less sandy with a fairly neutral pH (Table 3).

**TABLE 3:** Characteristics of the soils used in the pot experiments with the respective sites where the soils were collected.

Soil origin	Texture	Clay (%)	Fine silt (%)	Coarse silt (%)	Fine sand (%)	Coarse sand (%)	pH
Laon, France	Sandy-soil	10,1	6,6	10,6	65,6	7,1	7,9
La Gruche, France	Silty-sand	8,5	12,8	26	8,1	44,6	6,9
Gatteville-le-Phare, France	Sandy-clay	13,4	11,9	10,5	34,3	29,9	6,8

*Data across each raw with similar superscript lower case letters are not statistically different according to Tukey test (5% level of significance).*

#### Hatching of *Globodera pallida* in Soil

**FIGURE 3:**
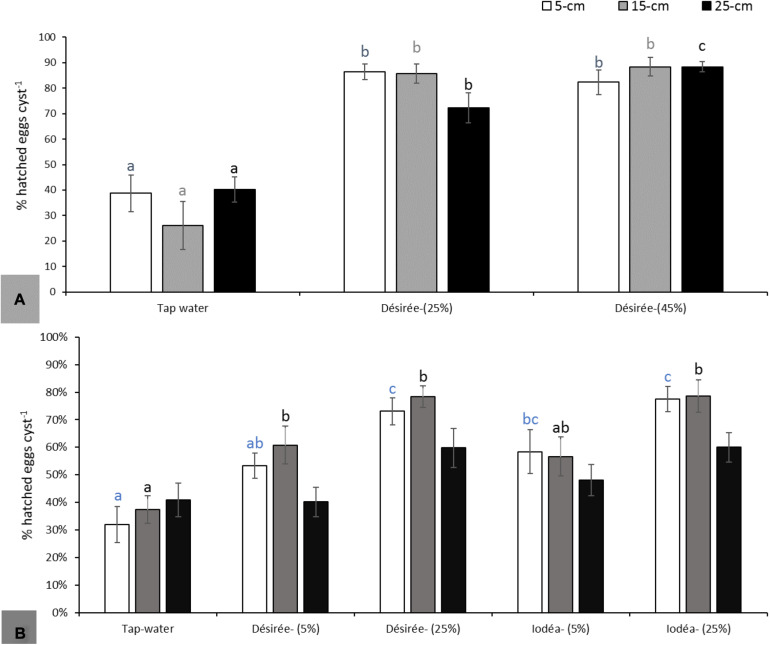
Hatching of *Globodera pallida* at 5, 15, and 25 cm soil depth following application of Désirée or Iodéa root exudates from top soil at 5, 25, or 45% concentration of the crude root exudates as compared with tap water in 2018 **(A)** and the repeated experiments in 2019 **(B)**. Different colored lowercase letters above the error bars represent significant differences between treatments for the same soil depth according to Tukey test (5% significance level).

Soil experiments conducted on *G. pallida* in 2018 revealed a significant increase of hatching (>70%) following potato cv. Désirée root exudate as compared with tap water applications (<40%) (*F*_2_,_27_ = 24.49; *P* < 0.001 at 5 cm, *F*_2_,_27_ = 32.2; *P* < 0.001 at 15 cm and *F*_2_,_27_ = 29.2; *P* < 0.001 at 25 cm; Figure 3A). However, there was no significant dose effect on the hatching rate with exudates diluted to 25 or 45%. There was also no significant depth effect on the rate of hatching for all concentrations of root exudates tested. Results obtained in 2018 were partially confirmed in the repeated experiments in 2019, but there were no significant differences at 25 cm soil depth (*F*_4_,_45_ = 1.872; *P* = 0.132; Figure 3B). For cysts placed at 5 and 15 cm soil depth, hatching was significantly higher than the control (tap water) for pots drenched with Désirée and Iodéa root exudates (*F*_4_,_45_ = 9.524; *P < 0.001* at 5 cm and *F*_4_,_45_ = 7.916; *P < 0.001* at 15 cm; Figure 3B). Whatever the root exudate (Désirée and Iodéa), hatching was higher at 25% as compared with 5% concentration of root exudates. In line with some observations in 2018, cyst incubated within the top 15 cm soil depth in 2019 experiments had an increase in level of *in situ* hatch as compared with hatching at 25 cm soil depth for *G. pallida*.

#### Hatching of *Heterodera carotae* and *H. schachtii* in Soil

**FIGURE 4:**
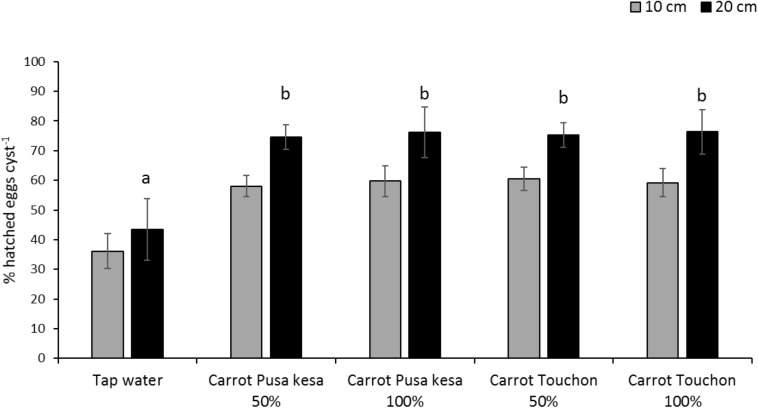
Hatching of *Heterodera carotae* at 10 or 20 cm soil depth following application of root exudates from *Daucus carota* cv. Pusa kesa or Touchon from top soil at 50 or 100% concentration of the crude root exudates as compared with tap water. Different lowercase letters above the error bars represent significant differences between treatments at the same soil depth according to Tukey test (5% significance level).

The general average hatch of *H. carotae* in soil drenched with root exudates of *Daucus carota* cv. Pusa Kesar or carrot Touchon was approximately 70% irrespective of the concentration of the root exudates, whereas in soil drenched with tap water, the hatching level was approximately 40% (Figure 4). At 20 cm soil depth, there was approximately a 15% improvement in hatching when compared with hatching of encysted eggs placed at 10 cm soil depth for all treatments with root exudates, irrespective of concentration. Significant differences between treatments and control were observed at 20 cm (*F*_4_,_45_ = 7.363; *P < 0.001*) but not at 10 cm (*F*_4_,_45_ = 2.018; *P = 0.108*) soil depth (Figure 4).

**FIGURE 5:**
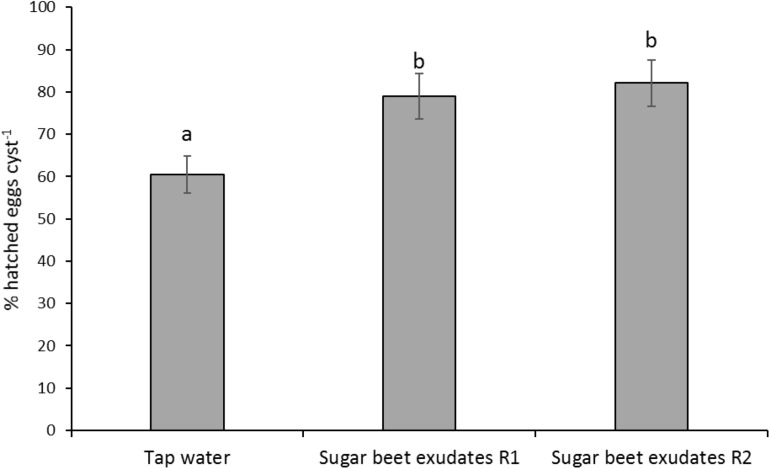
Hatching of *Heterodera schachtii* at 4 cm soil depth following application of 15 or 30 ml root exudates of sugar beet from top soil at 100% concentration at 2 (R1) or 4 (R2) days intervals, respectively, as compared with tap water as controls. Different lowercase letters above the error bars represent significant differences between treatments according to Tukey test (5% significance level).

*H. schachtii* performed well in pots drenched with tap water, inducing over 60% hatch, which was further significantly enhanced in pots drenched with undiluted sugar beet root exudates (*F*_2_,_27_ = 15.94; *P* < 0.001; Figure 5).

## Discussion

The main objective of this study was to evaluate the feasibility of the management of the three cyst nematode species, *G. pallida*, *H. carotae*, and *H. schachtii* using naturally produced root exudates. This study has provided some new and sometime surprising insights into the hatching behavior such as induction time, hatching dynamics and soil depth effects for the three studied cyst nematode species. Under the framework of optimizing the ‘suicide hatching’ strategy with natural plant secretions, this study enabled the development facilities for the mass production of root exudates with satisfactory levels of hatching induction attributes and the ideal conditions for application to obtain optimum levels of hatching for two of the three studied nematode species (*G. pallida* and *H. carotae*).

*In vitro* screening confirmed documented information about the host range of each species. *H. schachtii* exhibited high levels of hatching with the majority of root exudates. This could in part be attributed to its limited dependency on hatching stimulants as this species has a wide host range and also know to hatch readily in water (Perry, 2002). It was further demonstrated in soil experiments that favorable soil conditions (i.e., appropriate temperature and moisture) without host root exudates were sufficient to induce hatching of up to 60% in *H. schachtii*. However, hatching factors acted as accelerators in hatching dynamic as after 2 days of exposure, the core plants within its host range such as sugar beet, oil seed rape and other Brassicaceae induced hatching above 50%. With the observed levels of hatching without the presence of host root exudates, it was deemed not necessary to advice for the incorporation of the ‘suicide hatching’ strategy in the management of *H. schachtii*. This was because the application of root exudates would not generate substantial gains when compared with natural conditions only or the use of resistant varieties/cultivars or traps in an intercrop.

Unlike *H. schachtii*, the hatching of the carrot cyst nematode *H. carotae* was mainly induced by carrot varieties (*Daucus carota*). However, the novel findings included five wild relative species that featured within the top ten varieties, inducing high levels of hatching. Plants belonging to other genera outside *Daucus* were unable of inducing significant levels of hatching as compared with water. These observations are in line with the referenced narrow host range of this nematode species (Aubert, 1986) with a high dependence on chemical cues emitted by the host plant for hatch induction. This reinforced the hypothesis that *H. carotae* had coevolved with wild relative of carrots for a long time in its native area before being adapted to the cultivated varieties (Gautier et al., 2019).

The hatching behavior of *G. pallida* has been extensively studied (see Perry, 2002; Masler and Perry, 2018 for reviews) and not surprisingly, our screening revealed a strong induction of hatching by solanaceous cultivated plants such as potatoes, tomatoes and eggplants. The top five plants that induced the highest levels of hatching included three European cultivated varieties of potato, one cultivated variety of eggplant and also one wild potato from South-America. Nevertheless, the distinction between host and non-host for *G. pallida* did not appear as clear as for *H. carotae*, with a stable decline from 95% in hosts to 1% hatching in non-host species (Table 1A). However, the screening experiment revealed some surprising results with broccoli (*Brassica oleracea* var. Italica) which induced 56.7% hatch. This was unexpected as this plant has not previously been listed in *G. pallida*’s host range, and more so, the Brassicaceae are phylogenetically far from Solanaceae. These results raised the questions whether all broccoli varieties have this ability and whether hatching is stimulated at the same level for all populations of *G. pallida* [i.e., European population and South American populations belonging to the 5 clades described by Picard et al. (2007)]. These results revealed that, it might be possible to identify non-host plants that can induce substantial hatching and that it may be interesting to screen outside the referenced host range for future use as cash and/or trap crops. The identification of specific chemical cues present in broccoli root exudates might help save time usually spent on screening assays. This would facilitate the selection for appropriate cultivated plants and varieties based on their chemical composition as well as to compare the hatching factors present in broccoli root exudates with those present in solanaceous plants.

Following the mass screening, root exudates that produced satisfactory results under *in vitro* conditions were further assessed for ideal doses and exposure times necessary to induce optimum hatching, before the confirmation of their efficiency in a more complex soil environment. The choice was driven by the ability of the root exudates to induce satisfactory levels of hatching, as well as the ease of production. Two varieties of potatoes, Désirée and Iodéa were therefore selected for potato cyst nematodes, while two varieties of carrots, Touchon and Pusa Kesar were selected for carrot cyst nematode, *H. carotae* and two sugar beet varieties, Julietta and Acacia were retained for *H. schachtii*.

*In vitro* experiments demonstrated that simulating the presence of the host plant by exposing *H. carotae* or *G. pallida* to their respective host root exudates at very low concentration or for very short and repeated periods induced significant hatching as compared with water control. These observations are in line with previous reports, which demonstrated that hatching factors were able to induce substantial levels of hatching in cyst nematodes at very low concentration (Masler and Perry, 2018) and that a brief exposure may as well induce similar levels of hatching as a continuous exposure. There exist sufficient evidence of the hatching behavior for *G. pallida* (Perry and Beane, 1982; Perry, 2002), but little information was available on the hatching behavior of *H. carotae*. An average exposure time of 15 days to root exudates was necessary to induce satisfactory levels of hatching for both *H. carotae* and *G. pallida* with approximately over 60 and 80% hatched juveniles, respectively. For *G. pallida*, the level of hatching was comparable with hatching observed in continuous exposure to root exudates. This confirmed previous observations where 80% of encysted J2 hatched within 12 days of exposure (Masler et al., 2008a, b), which is an adaptation by infective juveniles to maximize their chance for successful infection of young healthy roots of the host plant (LaMondia and Brodie, 1984). It was also observed that brief exposure to root exudates for 6 h every 4 days was able to stimulate similar level of hatching as in continuous exposure. The dilution of potato root exudates to concentrations as low as 10% of the crude root exudates induced satisfactory levels of hatching, which was not significantly different from hatching in root exudates diluted to 25%. Preliminary experiments conducted on *H. carotae* exhibited similar hatching behavior (data not shown), suggesting that the high sensitivity to hatching factors, coupled with the high specificity were probably shared traits between species that had a narrow host range, such as *H. carotae* and *G. pallida*.

As a follow-up from *in vitro* studies, it was necessary to assess the performance of the root exudates in a more complex soil environment, much closer to field conditions. Therefore, treatments for the pot experiments were based on observations from *in vitro* tests. Observations on *H. schachtii* revealed only a slight increase in hatching percent for treatment with root exudate as compared with water, confirming *in vitro* observations. On the contrary, *H. carotae* and *G. pallida* responded better to host root exudates as compared with water, depending on the soil depth and on the applied dose of the root exudates. Generally, hatching is optimized at soil moisture equivalent to field capacity and favorable temperatures (Masler and Perry, 2018) which matches the conditions employed in the present study (within 60–80% of field capacity, at 14–20°C). There was a significant dose effect for Iodéa root exudates on *G. pallida*, but not for Désirée root exudates. Likewise, concentration was not an important factor for *H. carotae* under soil conditions. For *G. pallida*, hatching was often greater within the top 15 cm of soil than at 25 cm depth, except at 45% concentration in which the level of hatching was identical between the three soil depths investigated. The low hatch observed at 25 cm depth meant either that the root exudates did not reach the 25 cm level or that the quantity that attained this level was insufficient to stimulate the levels of hatch observed within the top 15 cm soil depth. These observations are in line with those of Ryan and Devine (2005) who demonstrated a progressive increase in PCN hatch over a period of 8 weeks post-planting, depending on the distance at which encysted eggs were incubated from the host plant. Earlier report by Rawsthorne and Brodie (1987) also demonstrated that root exudates produced near the root tip were biologically more active in hatching induction. However, unlike *G. pallida*, hatching was enhanced for *H. carotae* cysts incubated at 20 cm than for cysts incubated at 10 cm soil depth, with no observed dose effect.

From the present study, useful information could be drawn for future use of the ‘suicide hatching’ strategy for application on a field scale to control populations of *G. pallida* and *H. carotae*. This would involve the application of host root exudates: (i) in the absence of host crops, (ii) at a period where abiotic conditions will be favorable to hatching, (iii) at different intervals within the first 15 days or using a slow diffusion process (for granular forms) to stimulate encysted eggs to ensure optimum hatching and (iv) at a dose that allowed the induction of hatching within the vicinity of soil where cysts are usually detected (top 30 cm). These results would complement previous studies to address different challenges and select best practices. Devine and Jones (2001) investigated the effect of exogenously applied root exudates on hatching and in-egg mortality under field conditions, and although the effect of soil depth was not taken into account, the soil was rotavated following root exudates application to ensure their uniform distribution within the targeted soil profiles. Been and Schomaker (2013) reported approximately 90% of the PCN population within the upper 35 cm of soil in an infested field, while sampling at 25 cm was more uniform and represented approximately 84% of PCN population. Whitehead (1977) reported similar results for the vertical distribution of PCN, beet and pea cyst nematodes, although pea cyst nematodes were rarely found beyond 20 cm soil depth. The respective depth reported for each group of nematodes represents the vicinity within which the majority of host roots reside within the planting ridge. Under natural conditions, factors such as soil temperature, moisture, pH, organic matter content all influence the activity of hatching related compounds as well as the response of cyst nematodes to hatching chemicals (see Masler and Perry, 2018 for a review). Hatching activity induced by exogenously applied root exudates have previously been demonstrated to respond best and faster in sandy soils when compared with clay and peaty soils under field conditions (Devine and Jones, 2001). Poor results in peaty soils may in part be associated with possible sorption of hatching chemicals to organic matter, as negative correlations have previously been reported between organic matter content and hatch factors activity (Devine et al., 2001). In the present study, sandy soils were used for all groups of nematodes, and gave satisfactory results.

In addition to the influence of these parameters, major challenges will be (i) to ensure that soil biological communities have no impact on efficiency of hatching factors, or if it will have to be consider by adjusting the doses for instance, and (ii) to chemically identify hatching factors for both species for mass production and at a lower cost which would compensate for deregistered chemicals previously used in controlling these groups of cyst nematodes. Field validation of these observations are now needed.

## Data Availability Statement

The raw data supporting the conclusions of this article will be made available by the authors, without undue reservation.

## Author Contributions

All experimental protocols were jointly discussed and developed by the CMI, FN3PT/inov3PT, INRAE, and SILEBAN scientific and technical team. BN, MI, and PD established and assessed the laboratory and glasshouse experiments involving *Globodera pallida* conducted at FN3PT/inov3PT, and supervised by VG, AB, and A-CLR. NM, M-CD, CaP, and ChP established and assessed the laboratory and glasshouse experiments involving *Globodera pallida* and *Heterodera schachtii* conducted at IGEPP, and supervised by JM and SF. ER and AC established and assessed the laboratory and glasshouse experiments involving *Heterodera carotae* conducted at SILEBAN. Host root exudates for potato and beet cyst nematodes were produced at FN3PT/inov3PT, and IGEPP, respectively. Carrot root exudate production was jointly developed by SILEBAN and CMI, performed at SILEBAN, and supervised by ER and AC. Root exudate analyses was done at CIM Groupe-Roullier and coordinated by EN-O and J-CY, who also supervised the analysis for soil particle size distribution for the soils used in glasshouse experiments. All authors contributed to the article and approved the submitted version.

## Conflict of Interest

The authors declare that the research was conducted in the absence of any commercial or financial relationships that could be construed as a potential conflict of interest.

## Footnotes

1 Assessments conducted on *H. schachtii* only due to it fast rate of hatching.
